# Mixed method evaluation of a novel seminar format for a PBL-based integrated curriculum

**DOI:** 10.1007/s40670-026-02708-5

**Published:** 2026-04-09

**Authors:** Uliya Gankande, Felicity Roux, Denise Demmer, Vetri Thirthar Palanivelu

**Affiliations:** Curtin Medical School, Perth, Australia

**Keywords:** Flipped learning, Preclinical learning, Problem-based learning, Seminars, Blended learning

## Abstract

Medical students in the early preclinical years may find the depth of knowledge demanded for basic and clinical sciences challenging. Self-directed learning however necessitates identifying the depth of knowledge required. While multimedia depiction of the sciences may enhance learning, they are not tailored to the learning objectives for any given year curriculum. This study explored students’ experiences of a modified seminar format learning called “nCuSP”. It encapsulated a flexible blended learning experience using digital technology, and provided specific content to meet learning objectives. A mixed methodology was used. The first quantitative phase measured participants’ experiences of nCuSP and usual seminars. The second qualitative phase used focus group discussions to probe participants’ learning experiences. There were 356 responses from first- and second-year students. Their consensus was that the nCuSP format led to improved overall learning (80.1% vs. 19.9%). When contrasted with usual seminars, nCuSP led to better understanding of key concepts (67.550 *p*<.001) and knowledge consolidation (75.073 *p*<.001). Participants’ seminar preparation improved with nCuSP compared to usual seminars (89% vs. 8%), including higher peer interaction (93.2% vs. 6.8%) and problem-solving skills (88% vs. 12%). There were four focus group discussions with 14 participants representing both first and second years. Four main themes were identified: *overwhelm*, *prior knowledge*, *interaction*, and *time*. Findings suggest that the nCuSP seminar format based on blended learning using digital technology is a highly appreciated pedagogical approach in a module-based PBL-driven undergraduate medical education program.

**Supplementary Information:**

The online version contains supplementary material available at 10.1007/s40670-026-02708-5.

## Introduction

Modularisation of the early preclinical years of medical education with simultaneous provision of basic and clinical sciences is an established strategy to deliver an integrated preclinical curriculum [1]. While this process may decrease students’ cognitive load by focussing on clinically oriented learning, the task of curriculum developers and deliverers remains one of balancing an adequate knowledge of basic sciences with appropriate orientation to clinical based learning.

Especially in the early preclinical years, students may find the depth of knowledge demanded for both basic and clinical sciences challenging. For undergraduate programs particularly, school-leaver students face likely further challenges such as less-developed problem-solving skills [2] and reduced metacognitive skills [3] whilst also navigating the transition from adolescence to adulthood.

Preclinical medical education has progressed to using an integrated problem-based learning (PBL) curriculum. Given this, medical schools aim to identify and balance the depth of knowledge required in each discipline to support PBL. Much innovation has occurred with teaching modalities. For example, lectures have evolved to be delivered as recorded content [4] which increasingly replace in-person lectures. There is also a plethora of free commercial resources available online. In addition, there are 3-D virtual anatomy models and multimedia depictions of basic sciences to enhance learning.

However, these approaches are not sufficiently aligned with curriculum-specific learning objectives (LOs) and year level expectations. Despite the proliferation of digital resources which allow for increased student flexibility, seminars as key learning activities have had little innovation and remain predominantly didactic and lecturer-centred, characterised by passive transmission of information and limited opportunities for student interaction [5]. Evidence from medical education research indicates that passive learning structures are associated with diminished attention and cognitive engagement from students [6, 7]. These approaches often involve a one-way flow of information, insufficiently structured peer to peer learning opportunities, uniform pacing and dull content transmission. Combined with the lack of immediate feedback, these shortcomings fail to meet diverse learner needs and they constrain the development of the higher-order cognitive skills needed in medical practice. In contrast, blended formats foster active learning processes, deeper understanding and enhanced student engagement [6, 7]. 

To effect change requires recognising that the method of delivery impacts student learning [8]. In addition, best practice incorporates the higher orders of Bloom’s taxonomy [9] from recollection to analysis and application. Furthermore, the learning experience is enhanced when active learning modalities such as peer-to-peer discussions and interaction with subject specialists are added to a blended learning component [10].

Curtin University's pre-clinical medical student feedback received earlier as part of our continuous program improvement was clear: the usual seminar format was criticised for the poor interaction between guest-speaker presenters and students. It was considered disheartening for both these stakeholders, and there were concerns about the impact on student learning, engagement and attendance. Confronted with these pedagogical constraints; an obligation to meet accreditation standards; student demands for flexibility; and student expectations of embracing digital technology – the obvious task was to modernise our usual seminars to extend beyond these limitations.

We therefore modified the usual seminar format with a flexible and blended-learning structure, named the novel Curtin Seminar Praxis (nCuSP). It was purposively aligned with the PBL cases of the curriculum to guide students in their structuring of knowledge. Based on the core principles of blended learning theory [11], its aim was to create and trial a student-centred, flexible and interactive learning experience in which the student is an active participant. Using the key assumptions of the theory of constructivism [12], nCuSP allowed the students to build their learning experience using their existing knowledge and peer interactions. This study’s objectives were met in two phases:

1. The quantitative phase described students’ experience of nCuSP; and
2. The qualitative phase listened to the student voice as primary stakeholders in their own learning.

These findings may inform further research regarding blended learning and flipped classrooms and contribute to the evidence for undergraduate medical education.

## Materials and Methods

### Novel Curtin Seminar Praxis (nCuSP) Seminars

**Fig. 1:**
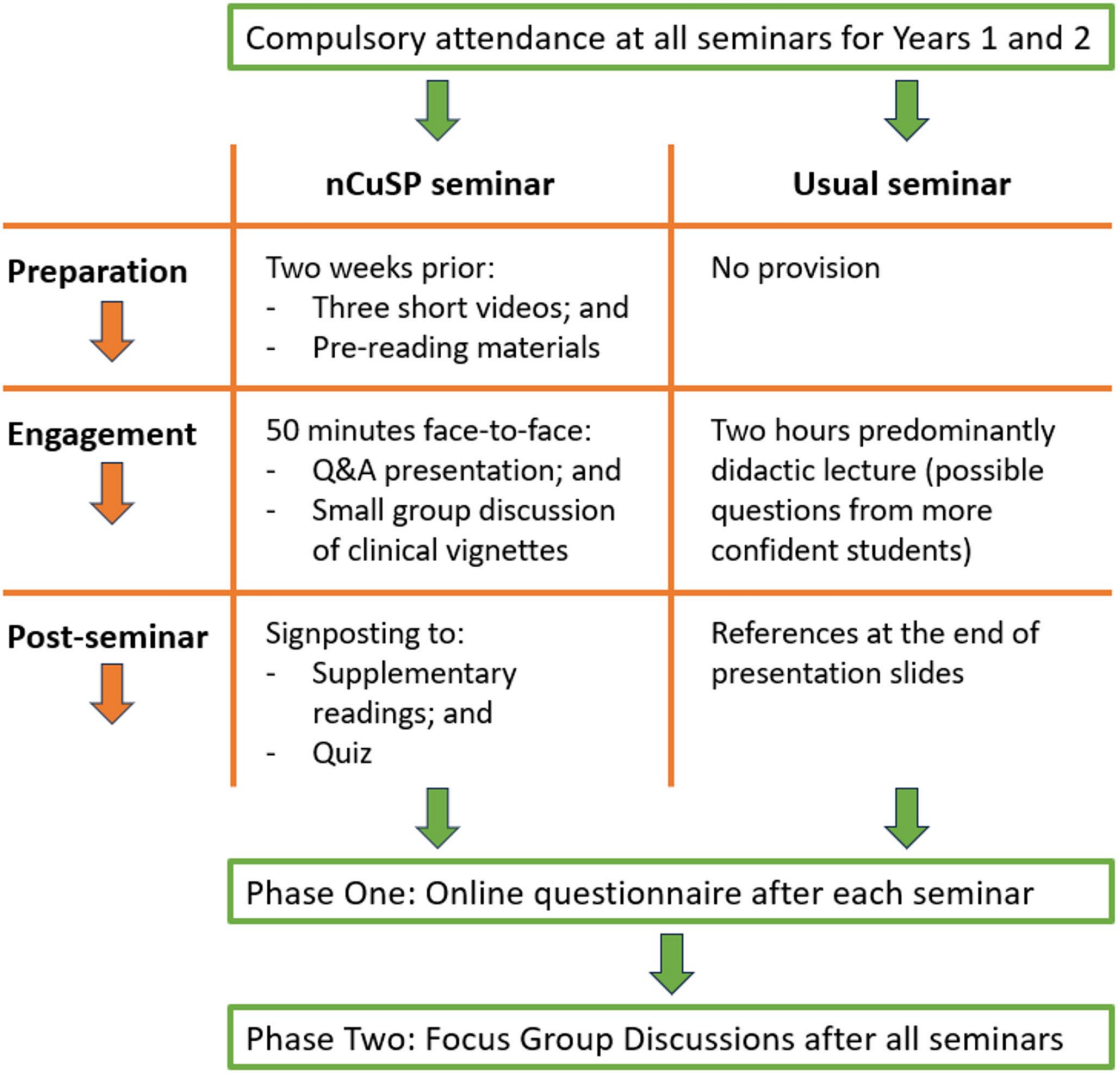
Study design and Comparison between the novel Curtin Seminar Praxis (nCuSP) seminars and usual seminars

Seminars are typically scheduled and delivered separately but within the context of PBL sessions. The nCuSP seminars are in a modified seminar format which encapsulated flexible blended learning, digital technology and flipped classroom techniques. The topic and the learning foci of each nCuSP seminar were matched to the current PBL case of the designated teaching week. The topics were analysed to identify elements that were suitable for theoretical content; development of polls and multiple-choice questions (MCQs); clinical vignettes; interactive components; and recommended readings. These elements were embedded across the two parts of each nCuSP seminar, consisting of a pre-nCuSP engagement followed by a face-to-face nCuSP seminar. Figure 1 compares the novel components of the nCuSP seminars to the usual seminars.

Each pre-nCuSP engagement was an online resource package. It was made available to the students through their learning management system two weeks before the face-to-face nCuSP seminar. It was estimated that students would spend approximately one hour engaging with the pre-nCuSP content. The resource package was comprised of two components. The first component was three short videos (of six minutes) which were created by the tutors who presented theoretical content in the usual seminars. Their duration respected the recommended optimal time of ≈ 20 min [13]. Each video contained interactive components such as embedded MCQs for enhanced engagement. This mirrors recent initiatives to incorporate interactive elements in between recorded chunks of lecture content [14]. The second component was two pre-nCuSP readings which were selected for their relevance to the LOs of the PBL case.

The subsequent face-to-face nCuSP seminar consisted of two components. The first component was a 20-minute PowerPoint presentation to the whole group. It reviewed key concepts and tested students’ understanding via an interactive Question & Answer with the guest-speaker presenter using Mentimeter [15]. The second component then divided the students into groups of ≈10 to facilitate peer-based discussion for 30 minutes. Each group was given two or more clinical vignettes, which were based on the seminar topic. The students discussed these vignettes within their small group. The guest-speaker with one or more seminar tutors circulated around the small groups to guide the students’ discussions. The face-to-face nCuSP seminar concluded with a summary of the clinical vignettes to the whole group, a short take-away message, signposting to supplementary readings and a post-seminar quiz.

### Study Design

In Semester Two of 2023, two seminars in both first and second years were delivered in the nCuSP format along with two usual seminars in the format of a two-hour didactic lecture (see Fig. 1). This mixed-methods descriptive study consisted of two sequential phases. Figure 1 describes the study design. In Phase One, a quantitative cross-sectional questionnaire was administered. In Phase Two, a qualitative study examined the responses from the questionnaire’s open-ended questions with focus group discussions (FGDs). Curtin University Human Research Ethics Committee approved this study (approval HRE2023-0374).

### Participants

A purposive sampling strategy was used in Phases One and Two to recruit participants. The target population comprised of preclinical students, which consisted of both first and second years (*N* = 224). No distinction was made between these two years. The sampling frame was derived from class lists. The inclusion criteria were current admission to Curtin University, enrolment in its medical program at the preclinical level, and seminar attendance. Therefore, all students from the first and second years were eligible and invited to participate.

Two information sessions were offered one month prior to the study’s commencement. All students were given hard copies and a portable document format of the Participant Information Statement. Students’ participation was voluntary. Participants provided their consent online if they chose to answer the questionnaire. However, written consent was obtained from participants contributing to a FGD. Specifically, responses were anonymous for the survey of Phase One, but not for the FGDs of Phase Two.

#### Phase One: Quantitative Data Collection and Analysis

The questionnaire was based on one used for Curtin Medical School's internal evaluations with which students were already familiar, and enhanced with reference to the literature [8, 16]. It consisted of two 5-point Likert scaled question items, whereby the second Likert scale was only applicable to to nCuSP seminars. Items such as ‘Viewing videos was a better experience than usual seminars’ and ‘I had sufficient guidance to complete the clinical vignette’ pertained solely to the nCuSP seminar format, As detailed in Fig. 1, these features were absent from the usual seminars. The non-existence of these features in usual seminars therefore meant it was not possible to directly compare nCuSP with the usual seminars because students formed their opinions about nCuSP based on their previous experiences with usual seminars. The if-then skip logic functionality of the questionnaire ensured that responses were relevant for the usual seminars. In addition, there were two open-ended questions and four selectable close-ended questions which applied to both seminar types. The questionnaire is included in Supplementary Materials. At the end of each seminar, consenting participants completed the online questionnaire.

Likert responses were categorised into “Agree”, “Neither agree nor disagree” and “Disagree” categories. Descriptive questionnaire data are shown as frequencies and percentages. Data were analysed using the Statistical Package for the Social Sciences [17]. Both descriptive and inferential statistics (Pearson’s χ2 test and Fisher’s exact test) at a significance level of 5% were generated. While the chi-squared test and Fisher’s exact test can be used when comparing seminar types, the Fisher’s exact test was preferred over the approximation assumed by the Chi-squared test in this instance [18]. For each category, proportions were calculated and reported with 95% confidence intervals, which were estimated using Wilson’s score method for binomial proportions to provide robust interval estimates for categorical data with unequal group sizes and small cell counts.

#### Phase Two: Qualitative Data Collection and Analysis

There were two sources of qualitative data. The first was derived from the questionnaire’s anonymous open-ended responses. Each response was linked to participants’ year and seminar. Responses were thematically categorised.

The second source used the COREQ checklist [19] to guide the FGDs. After all questionnaires were completed (Phase One), students were invited to participate in a face-to-face FGD on campus at their convenience for 50 minutes. These were conducted by FR, an experienced facilitator. Participants were encouraged to reflect honestly on their learning experiences of both nCuSP and usual seminars. To minimise the potential bias of self-selection, participants were assured of anonymity in order to reduce any influence of their perceived expectations of answers. This was achieved through FR’s role in blinding participants’ identities to UG, DD and VTP, which was facilitated because FR had no role in assessments or course progression decisions. The FGDs were audio-recorded and transcribed verbatim by a reputable external agency. Pseudonyms replaced participants’ names.

Reflective thematic analysis enabled descriptive and interpretative accounts of the data [20]. This involved searching and identifying common threads [21] which extended across the open-ended responses from the questionnaire and the FGDs’ transcripts.

Analysis began with data familiarization by rereading the FGDs’ transcripts with the audio-recordings to ensure accuracy and de-identification. The cleaned transcripts and open-ended responses were then imported into NVivo^®^ [22] and systematically coded line-by-line by FR. Two randomly selected de-identified clean transcripts were independently coded by UG. Their codings were compared and rechecked against transcripts to maintain data dependability. Preliminary interpretations were discussed within the team. Consensus was reached after a continual sorting and coding of data into meaningful patterns [23]. Further analysis of these patterns facilitated the naming of themes and subthemes [24].

Bias and confirmability were addressed by constant comparative data analysis, which facilitated understanding and interpretation [25]. Bias was reduced through the iterative process of re-evaluating the categorisation of emerging data rather than prematurely fixing initial assumptions. Confirmability was enhanced because constant comparison refines interpretations to fit the data, thus reducing arbitrary conclusions.

## Results

### Phase One: Quantitative Results

The average response rates across all seminar types were 56.71% for Year 1 and 23.69% for Year 2. Table 1 describes the response rate by year group and separated by the seminar type.

**Table 1:** Responses rates by year group and separated by type of seminar

Year	nCuSP Seminars *n* (%)	Usual Seminars *n* (%)	Total
Year 1	nCuSP–Y1–#1	Usual–Y1–#1	
*N* = 110/224	69 (62.73%)	55 (50.00%)	
	nCuSP–Y1–#2	Usual–Y1–#2	
	72 (65.45%)	52 (48.64%)	
Total responses	141 (56.85%) (Mean rate 64.09%)	107 (43.15%) (Mean rate 49.32%)	248 (100%) (Mean rate 56.71%)
Year 2	nCuSP–Y2–#1	Usual–Y2–#1	
*N* = 114/224	46 (40.35%)	27 (23.68%)	
	nCuSP–Y2–#2	Usual–Y2–#2	
	10 (8.77%)	25 (21.93%)	
Total responses	56 (51.85%) (Mean rate 24.56%)	52 (48.15%) (Mean rate 22.81%)	108 (100%) (Mean rate 23.69%)
Grand total of responses	197 (55.33%)	159 (44.67%)	356 (100%)

*nCuSP* novel Curtin Seminar Praxis

Nearly all dimensions explored in the questionnaire demonstrated a preference for the nCuSP seminar format (see Table 2). The overall learning experience of the nCuSP seminar was highly statistically significant (75.699 *p*<.001). Participants’ understanding of key concepts was moderately statistically significant (67.550 *p*<.001) while knowledge consolidation is highly statistically significant (75.073 *p*<.001).

**Table 2:** Comparison of the novel Curtin Seminar Praxis (nCuSP) seminars and usual seminars

Question	Seminar type nCuSP (*n* = 197) Usual (*n* = 159)	Agree *n* (%) [95% CI]	Undecided *n* (%) [95% CI]	Disagree *n* (%) [95% CI]	Fisher-Freeman- Halton Exact Test *p*-value
Confidence to interact	nCuSP	171 (86.8) [81.4–90.8]	16 (8.1) [5.1–12.8]	10 (5.1) [2.8–9.1]	50.413
	Usual	86 (54.1) [46.3–61.6]	30 (18.9) [13.5–25.7]	43 (27.0) [20.7–34.4]	< 0.001
Adequate tutor feedback	nCuSP	168 (85.3) [79.7–89.6]	27 (13.7) [9.6–19.2]	2 (1.0) [0.3–3.6]	43.829
	Usual	86 (54.1) [46.3–61.6]	60 (37.7) [30.6–45.5]	13 (8.2) [4.8–13.5]	< 0.001
Understanding key concepts	nCuSP	180 (91.4) [86.6–94.5]	11 (5.6) [3.1–9.7]	6 (3.0) [1.4–6.5]	67.550
	Usual	88 (55.3) [47.6–62.9]	25 (15.7) [10.9–22.2]	46 (28.9) [22.4–36.4]	< 0.001
Knowledge consolidation	nCuSP	176 (89.3) [84.3–92.9]	14 (7.1) [4.3–11.6]	7 (3.6) [1.7–7.2]	75.073
	Usual	78 (49.1) [41.4–56.8]	35 (22.0) [16.3–29.1]	46 (28.9) [22.4–36.4]	< 0.001
Critical thinking	nCuSP	168 (85.3) [79.7–89.6]	20 (10.2) [6.7–15.2]	9 (4.6) [2.4–8.5]	42.753
	Usual	88 (55.3) [47.6–62.9]	34 (21.4) [15.7–28.4]	37 (23.3) [17.4–30.4]	< 0.001
Studying time management	nCuSP	132 (67.0) [60.2–73.2]	35 (17.8) [13.1–23.7]	30 (15.2) [10.9–20.9]	47.500
	Usual	58 (36.5) [29.4–44.2]	25 (15.7) [10.9–22.2]	76 (48.0) [40.2–55.5]	< 0.001
Motivation to study	nCuSP	139 (70.6) [63.8–76.5]	39 (19.8) [14.8–25.9]	19 (9.6) [6.3–14.6]	44.672
	Usual	64 (40.3) [33.0–48.0]	37 (23.3) [17.4–30.4]	58 (36.5) [29.4–44.2]	< 0.001
Learning experience	nCuSP	170 (86.3) [80.8–90.4]	17 (8.6) [5.5–13.4]	10 (5.1) [2.8–9.1]	75.699
	Usual	69 (43.4) [35.9–51.2]	48 (30.2) [23.6–37.7]	42 (26.4) [20.2–33.8]	< 0.001

*CI* Confidence Interval

Values are *n* (%); percentages are presented with 95% confidence intervals calculated using Wilson’s score method

As shown in Table 3, participants clearly agreed that the nCuSP seminar format was superior to the usual seminar format in both recorded content and student preparation for seminars (89.0% vs. 8.0%). Similarly, when comparing nCuSP and usual seminar formats, the clinical vignette discussion participation was high (86.4% vs. 8.1%). Furthermore, Fig. 1 displays the participants’ consensus that nCuSP format led to an improved learning experience (80.1% vs. 19.9%).

**Table 3:** Participants’ description of the novel Curtin Seminar Praxis (nCuSP) seminars

Question	Agree *n* (%) CI	Undecided *n* (%) CI	Disagree *n* (%) CI
Recorded content	170 (89.0%) CI: 84.8–93.2	9 (4.7%) CI: 2.1–8.4	12 (3.3%) CI: 3.1–9.9
Preparation for seminar	170 (89.0%) CI: 84.3–93.2	9 (4.7%) CI: 1.6–7.9	12 (3.3%) CI: 3.1–9.9
Clinical Vignette participation	165 (86.4%) CI: 81.2–91.1	23 (6.5%) CI: 7.9–16.8	3 (1.6%) CI: 0.0-3.7
Questions prepared from pre-nCuSP materials	92 (48.2%) CI: 41.4–55.0	58 (30.4%) CI: 23.6–36.6	41 (21.5%) CI: 15.7–27.7
Tutor interaction	142 (74.3%) CI: 68.1–80.1	38 (19.9%) CI: 14.1–25.7	11 (5.8%) CI: 2.6–9.4
Peer to peer interaction	178 (93.2%) CI: 89.5–96.3	7 (3.7%) CI: 1.0-6.3	6 (3.1%) CI: 1.0-5.8
Peer to peer problem solving	168 (88%) CI: 83.2–92.7	17 (8.9%) CI: 5.2–12.6	6 (3.1%) CI: 1.0-5.8
Easier than usual seminars	130 (68.1%) CI: 61.3–74.9	42 (22%) CI: 16.2–28.3	19 (9.9%) CI: 5.8–14.1
This format improves learning	153 (80.1%) CI: 74.3–85.9	20 (10.5%) CI: 6.3–15.2	18 (9.4%) CI: 5.2–13.6

*CI* Confidence Interval

**Fig. 2:**
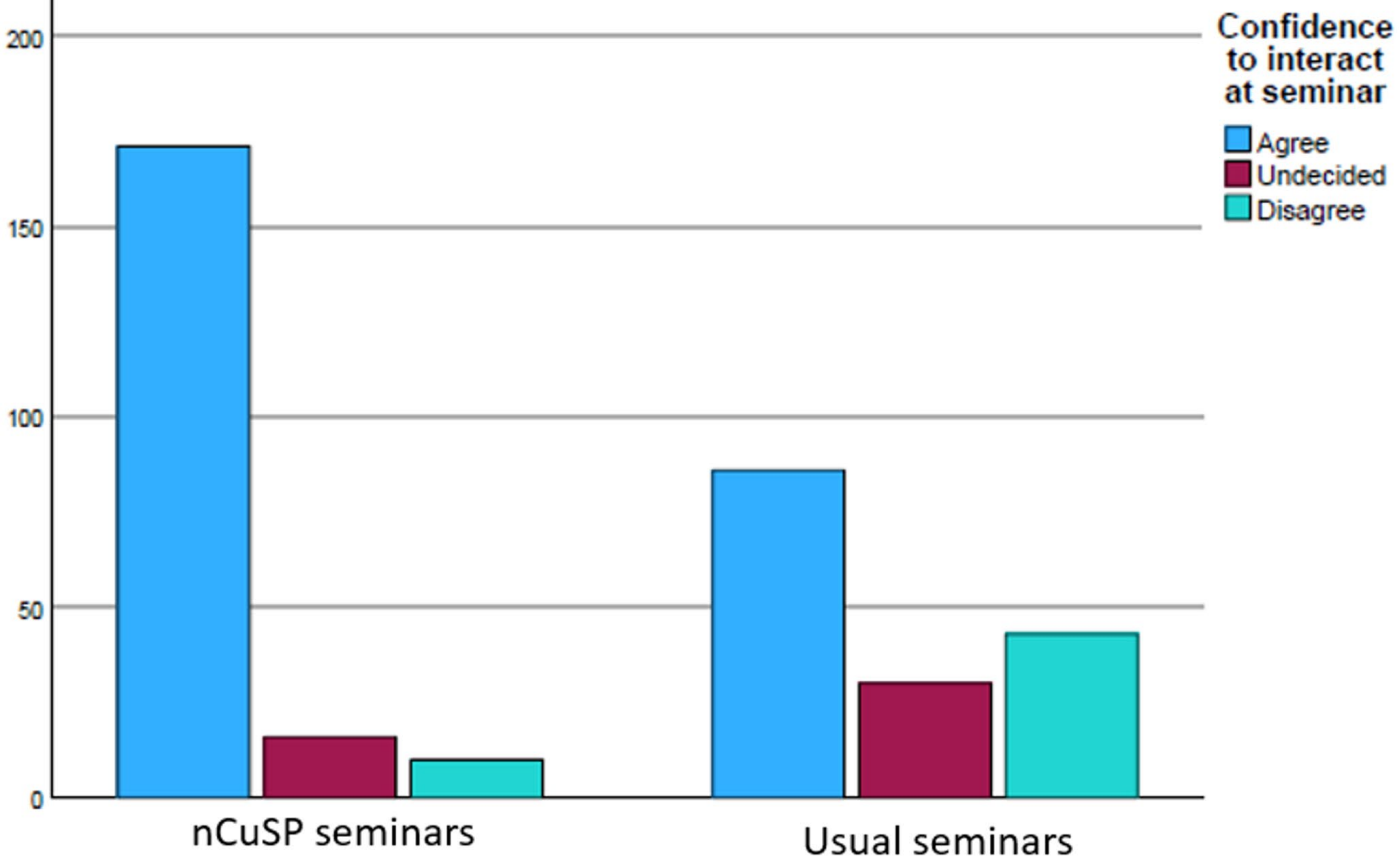
Confidence to interact at novel Curtin Seminar Praxis (nCuSP) seminars versus usual seminars

Figure 2 shows participants’ observation that peer interaction was exceptionally high in the nCuSP seminar format (93.2% vs. 6.8%). Similarly, they agreed that the nCuSP format improves peer-to-peer problem-solving skills significantly (88.0% vs. 12.0%). Whilst the participants were less likely to prepare questions using preparation materials (nCuSP 48.2% vs. usual 51.9%), they were more likely to interact with tutors during the nCuSP seminars (74.3% vs. 25.7%).

### Phase Two: Qualitative Results

There were four FGDs with 14 participants: two FGDs with Year 1 participants (*n* = 6) and two FGDs with Year 2 participants (*n* = 8). Four main themes were identified: *overwhelm*, *prior knowledge*, *interaction*, and *time*. These are presented in Table 4 with illustrative quotes of the 14 subthemes.

**Table 4:** Illustrative quotes of main themes and subthemes

	Main theme	Subtheme	No. refs	nCuSP seminars	Usual seminars
1	Overwhelm				
	1.1	Clear Learning Objectives	28	This course is very overwhelming. Whereas with these flipped seminars, it was very clear what exactly were the learning objectives we needed to know. (Y1Kate)	I found the lecture to be very theoretical and niche, and probably not important for our understanding of clinical practice or research. Yes, there are important concepts, but we have to learn so much material. (Y2UsualPopulation)
	1.2	Keeping up	13	I enjoyed being able to learn and make notes prior to the lecture using the pre-recorded seminar. This ensured that I was prepared with the content before the seminar, and was able to make thorough notes on the content (instead of having to rush to make notes in seminars). (Y1nCuSPEatDisorder)	I’m spending so much time trying to copy the words down that I can’t listen and take in everything, and then I don’t understand more stuff down the line. (Y2Jun)
	1.3	Watching	17	But if you need to work more slowly, pre-watching the nCuSP stuff, you can kind of go back or pause it, and finish your notes. Because, just given the nature of lectures, the lecturer will just keep talking. And just as you start to understand something, they click the slide, and it’s gone. (Y2Lia)	They [lecturers] always ask, “do you want me to power through or do you guys want a break?”, and everyone says, “power through” cos we’re like “let’s just get to the end because we’re going to rewatch it anyway”. (Y2Ruby)
	1.4	Zoning out	16	The discussion and questions meant I didn’t zone out as much and was more conducive to my learning (Y1nCuSPEatDisorder)	I sit at the back of the room and I can see most people’s laptops. Some are taking notes, but a lot of people are just online shopping or doing other stuff. (Y2Sarah)
2	Prior knowledge				
	2.1	Capacity	10	I really like receiving the content beforehand and having the opportunity to learn it. That way I can work on applying and consolidating the knowledge during the seminar. (Y2nCuSPHealing)	We only learnt about the stroke patient this morning. My knowledge was limited and a lot of us didn’t have the capacity to answer questions and contribute. (Y1UsualBrain)
	2.2	Digestible	22	I liked that we could view the seminar content in our own time, giving us sufficient time to learn the content. It was then useful to apply what we learned in class with the tutor available if we had any questions. (Y2nCuSPHealing)	In a lecture, you’re getting thrown all this stuff. There’s words you don’t know. And then especially with me, I just lose track. Then there’s more pressure to go home and rewatch it. Yeah, I feel normal [usual] seminars are a lot more on me personally. (Y2Sarah)
	2.3	Motivation	15	By being able to learn the content and prepare for the seminar beforehand, it ensures students will learn the content even if they require leave. It also allows application of knowledge learnt, which greatly helps to consolidate learning and increase motivation and energy in the seminar room. (Y2nCuSPHealing)	This morning’s lecture was on carbohydrates, and you could probably count maybe 20 people. I mean, you’ve seen it, walking into a seminar and the place is a ghost town. That’s the proof of it, really. People sort of accept defeat and just leave. (Y2Lia)
	2.4	Shame	20	When I go into lectures where I already know what’s happening and what’s being done, like I feel smart. So I’m going to be more likely to listen and interact and ask questions and stuff like that, yeah. (Y2Sarah)	It’s just a weird feeling, but I’d say 90% of the cohort is a bit anxious about asking questions. Yeah, I don’t know. It’s just like med students, we’re scared to show weakness. (Y1Megan)
3	Interaction				
	3.1	Tutor engagement	16	I like that the lecturer will come around to each table because having someone answer your questions is so much easier than checking Google. Google sometimes will give you stuff way out of the scope and sometimes you just need a simplified answer. (Y1Kate)	It’s very monotonous and long since it’s just us listening to a speaker. It’s very hard to concentrate and I find myself sleeping. (Y1UsualBrain)
	3.2	Peer group discussion	23	There is always someone who doesn’t understand. Then if you say your answer, they’ll ask you why and then you have to explain it, which then helps both of you in understanding it. (Y2Ruby)	In a lecture, people aren’t communicating. So you’re just in your own head with your own opinion, thinking that’s the right answer. But if it’s discussed, you hear someone say something completely different, and you can find out why they thought that. (Y1Charlotte)
	3.3	Active recall triggers	8	One person will say something, and then there’ll be someone who remembers another case. And you can see how our brains start working because you have all these different triggers coming from different points. It’s like active recall in a way (Y2Sarah).	With the usual seminars, it’s passive. Everyone’s always checking the time; some people are just clueless, and it’s always the same people asking questions. (Y1Megan)
	3.4	Applied problem solving	24	Having knowledge going in meant that we were able to go through clinical applications and questions which helped my problem-solving and to consolidate my understanding, giving me the ability to check my knowledge and fill any gaps. Y2nCuSPHealing	We’re not expected to apply anything in usual lectures. It would be: “this is how adrenal insufficiency works”. The end. (Y2Ruby)
4	Time				
	4.1	Efficiency	16	I can get through the content faster on my own and I can choose what I think is beneficial and not beneficial to my learning. Yeah, it’s cool to select what content you want to learn. (Y2Declan)	I think personally my lecture days are wasted days because I’m not taking it in. If I’m lucky, I’ll take in maybe one out of four lectures. The other ones will just be wasted time. So if I could do that at home, at my own pace and actually take things in, and then come in and discuss it with the class to solidify, that would be way more efficient. (Y2Ruby)
	4.2	Timing	23	When I watched the videos about wound healing, I was still interactive and focused in that lecture. Even though we had a PBL after that lecture, I still understood what was happening because of the videos. (Y2Sarah)	We’ve just done a whole week of thyroid learning in our own time. And now we have a lecture on Thursday about it, which will be great revision but some people might decide not to come into that because they’ve covered it already in their own time. (Y2Jun)

*nCuSP* novel Curtin Seminar Praxis

#### Theme One: Overwhelm

Participants reported that their experience of learning medicine is overwhelm. When asked to explain how seminars might help address this, the participants highlighted the importance of *clear learning objectives* (1.1). They found that nCuSP seminars were helpful for “knowing what I need to know” (Y1nCuSP-Fertility). In contrast to usual seminars, “it was unclear which aspects were important in terms of key takeaways” (Y1Usual-Bullying).

Participants expressed their *overwhelm* as a challenge of *keeping up* (1.2), for example:

There’s two or three students that really shine out, that always try and interact. And bless their souls, for some reason they’re so well-read obviously. But in 98% of the case, all of us are still trying to catch up. (Y1James)

The nCuSP seminar format may have removed this frustration because it required *watching* (1.3) the nCuSP videos beforehand. One participant observed, “it’s better if I can first watch it at home and then absorb it in the seminar” (Y1Kate). Given the struggle with *keeping up* (1.2) in usual seminars, one participant reflected that “often I’d end up having to re-watch the seminar anyways” (Y2Lia).

Participants candidly reported on managing *overwhelm* by *zoning out* (1.4). The difference between nCuSP and usual seminars was described as follows:

Yeah, if you’re sitting there alone just listening to someone, you can easily get zoned out or checking emails that pop up. And I find the interaction [with nCuSP] keeps my attention by being held accountable by the group, and you’re challenged. (Y1James)

#### Theme Two: Prior Knowledge

Overall, participants expressed their preference for *prior knowledge* when attending seminars. This increased their *capacity* (2.1) to engage, for example:

The good thing about the flipped seminar is that you come prepared with a base level of what you need to know. So if there is something you don’t understand, you come armed with questions. (Y1Harry)

This was facilitated by the nCuSP format providing *digestible* (2.2) tranches of information which gave “processing time which you don’t get in a normal [usual] seminar” (Y1Maxine). This ties closely with the *motivation* (2.3) to continue studying, as one participant explained:

You get to have the information at home to digest in your own time; and then you reason through it with your friends and you actually use that information. It’s not just sitting in a text box in your head. And then you feel happy with yourself; like you want to keep doing the degree because you’re actually doing good at it. (Y2Ruby)

Not having *prior knowledge* was linked with *shame* (2.4). As a Year 1 participant explained:

In order to get into medicine you have to strive to be really top dog. So I think we’ve that foundation that we shouldn’t be getting things wrong. So then by admitting that we don’t know something, it’s… I don’t know, just intrinsically wrong. (Y1Kate)

This was echoed by a Year 2 participant:

You feel like you’re stupid because it’s there on the board, but you don’t understand it. And then you just feel like crap because you don’t get it and it’s right there. I guess it’s kind of the shame of not understanding it. (Y2Sam)

Compared with usual seminars, nCuSP seminars gave students *prior knowledge*, which built their *capacity* to engage by providing *digestible* information to process in their own time. This may have helped assuage students’ *shame* and thereby improved their *motivation* to study.

#### Theme Three: Interaction

The nCuSP seminars introduced an element of *interaction*. This was highlighted by one participant who contrasted its *tutor engagement* (3.1) with the view that “I think one and a half hours of the tutor talking at us is a lot” (Y2UsualMidwifery). Additional interaction was with *peer group discussion* (3.2). This was a valued feature of nCuSP because “hearing from other students their reasoning was very helpful as it made certain concepts clearer for me” (Y1nCuSPFertility).

Participants suggested that the *interaction* was helpful because it engaged *active recall triggers* (3.3). One participant explained the process as follows:

Not everyone can remember everything. There’s some people who are lucky with brains like that. But each person seems to fill in each other’s gaps. And then when you discuss it together, you remember more. (Y2Ruby)

Participants appreciated how the *interaction* facilitated *applied problem-solving* (3.4). As one participant reflected, “the clinical vignette really helped us apply the knowledge we had just learned” (Y1nCuSPEatingDisorder).

#### Theme Four: Time

A common refrain was the scarcity of *time* when studying medicine as a content-heavy subject. Participants deliberated over *efficiency* (4.1), as follows:

The good thing about the usual format was that you obviously didn’t have to do any pre-work and that’s more time efficient. But, when it comes to exams, I would feel a lot more prepared with the flipped seminar compared to the usual. So it would save me more time in the long run. (Y1Megan)

The order in which usual seminars fit with their PBL cases highlighted an issue with *timing* (4.2) with usual seminars. As one participant explained:

The tutors are getting frustrated with our lack of interactivity, and that comes back to the timing and a lack of knowledge going in. So before we’ve even learned the LO’s of that week, they give us the content from the seminars, and then complain that we’re not interactive enough. Well, it’s because we haven’t learned anything. We have nothing to ask because we don’t know. (Y1Charlotte)

In contrast with nCuSP seminars, participants reflected, “I don’t think it [timing] really matters much” (Y2Declan).

## Discussion

### Interpretation

This study’s first objective was to describe participants’ experience of nCuSP. Whilst the response rate varied between the first and second years, both cohorts appreciated the modification of their usual seminar format. The nCuSP model delivered a satisfactory overall learning experience for students (75.699, *p*<.001) adding further evidence to an established positive effect of the flipped classroom model [26, 27]. These results indicate the success of this pedagogy in a module-based PBL medical course.

Participants reported that the nCuSP seminar model was superior to the usual seminar format for its recorded content and student preparation (89.0% vs. 8.0%). The success of the nCuSP model can be explained partially by self-determination theory [28]. This identifies autonomy, relatedness, and competence as the intrinsic needs which are central to motivation [28]. Encouraging self-directed learning likely orients medical students for continual learning within this profession, and marks a shift from rote learning [29].

The study’s second objective was to listen to the student voice as primary stakeholders in their own learning. Similar to current evidence [26, 27, 30], the participants valued the unrestricted access to videos which aligned with the specific LOs of their PBL cases, plus the availability of learning material to study at their own pace. The unique aspect of the nCuSP recordings was the embedding of active learning in the recordings to maintain engagement.

This study suggests that undergraduate students may perceive a lack of prior knowledge as shameful. Approximately 40% of Year 1 students in 2024 had not previously studied human biology. This likely made their first year challenging because of a heavy cognitive burden which may have caused overwhelm. Adhering to Bloom’s taxonomy [9], students are essentially confronted with new and challenging concepts, which they are required to apply within a short time frame. Admission to a medical degree is a highly competitive process, demanding academic excellence and social maturity. Recognition of this was related to the intervention design, whereby the pre-nCuSP engagement familiarised students with new key concepts which were subsequently refreshed in their memory during the face-to-face nCuSP seminar. Therefore, the provision of structured preparation material and supporting students to accentuate links with the PBL cases may alleviate overwhelm for these high achievers. Consolidating learning through this phased approach likely increased familiarity with the key concepts and therefore increased confidence.

The self-paced learning likely had a positive effect on the depth of PBL case discussions and may have led to better knowledge consolidation, which was statistically significant (75.073, *p*<.001). Both flexibility and autonomy of learning may in turn lead to increasing competence. From a pedagogical perspective, nCuSP may be helpful in steering students to the higher levels of Bloom’s taxonomy [9] earlier in their medical education. Under supervision of a subject specialist [31], the nCuSP seminar created a learning environment for the students to evaluate, further investigate evidence and construct their own understanding by engaging in hands-on clinical vignette activities. This represents a substantial departure from usual seminar methods.

Furthermore, there are implications for curriculum design beyond this study’s immediate context. Typically, the didactic lectures of the usual seminars are two hours’ duration, whilst the face-to-face contact time for the nCuSP is one hour. This timesaving could therefore be allotted to other learning activities, such as fieldwork or research projects.

In addition to sound pedagogical underpinnings and adaptation to the digital age, the nCuSP recordings offered the opportunity of building a useful repository of materials relevant to specific LOs. In time, this repository can be made available as students’ revision material; used in other year groups with specialist mediators to enhance face-to-face sessions; and viewed as a cost-effective resource of reducing the carbon footprint.

### What Makes nCuSP Different?

Within most medical curricula, seminars and lectures are important providers of primary knowledge as complimentary to parallel learning activities. This study’s findings are comparable to similar studies in medical education, specifically regarding a variety of learning activities supported by technology [32]; greater student engagement and satisfaction [33]; and enhanced knowledge comprehension [34].

However, the nCuSP model is unique. Firstly, interactive learning modalities are included for both online and face-to-face content. Secondly, students can engage with a subject specialist before, during and after the learning. The nCuSP format focused on enhancing module-based PBL driven medical education by using a combination of established pedagogical methods and weaving it with current technology for meeting the needs of the digital age. The nCuSP format aims to lay a foundation for scaffolded and supported self-learning within a module-based learning in a PBL curriculum.

Outside of medical education, this study’s findings correlate to studies in nursing [30] and pharmacy [15] where a similar pedagogy demonstrated an increased motivation among students and student preparedness. Likewise, within undergraduate engineering, student satisfaction [35] and improved student motivation, readiness and critical thinking skills [36, 37] were observed. Therefore, while this study is unique within a PBL-driven undergraduate medical education, its findings are comparable across other fields of higher education.

### What Makes this Research Study Unique?

To the best of our knowledge there are few studies that have used blended learning methods with a flipped classroom approach in a module-based PBL-driven medical program which has listened to the “student voice” [32]. Similar studies could be enhanced by students’ reflections [33, 34]. Therefore, this study offers additional insight into medical students’ learning processes and needs.

A recent study [33] has identified similar themes to nCuSP such as usefulness of pre-lesson material, greater engagement and overall satisfaction of the blended learning approach within medical education. However, themes such as overwhelm, shame and time pressures were not elicited. Therefore, this study provides new insight into aspects which are important considerations for learning institutions conscious of supporting medical students’ mental health and wellbeing. Our adaption of the blended learning approach to reduce the cognitive burden on students and provide flexibility is further validated by the literature [8, 38].

Directions for future research include longitudinal impact or scalability across different curricula, both medical and non-medical. Furthermore, investigation into insights from institutions for educator preparation are warranted.

### Strengths and Limitations

The strengths of this study include the use of mixed methods which adds validity to the design [39]. However, students participated voluntarily, conferring selection bias. Since attendance at seminars is compulsory, participants were able to answer for multiple seminars. This would have further increased selection bias. Tutor variations in teaching style as well as the content of flipped classroom sessions of the nCuSP seminars, compared to usual seminars, may have influenced the outcomes, thus suggesting framing bias.

Furthermore, the sample size was small. Consequently, achieving significance was not possible in presenting data by cohort for the purpose of facilitating comparison and identifying year-specific patterns.

In addition, this study was conducted in a single-institution context. Therefore, its results may not necessarily be generalisable, particularly in schools with a graduate medical program or with a different student demographic.

Finally, this study relied solely on student surveys and FGD-based feedback, which renders the conclusions subjective. Future studies would include an objective indicator of student performance, such as assessment scores, skill evaluations or competency measures.

## Conclusion

The overall learning experience of the nCuSP seminar is significantly superior (75.699 *p*<.001) to the usual seminar format. Therefore, the nCuSP seminar format based on blended learning using a flipped classroom model with digital technology is a highly appreciated pedagogical approach in a module-based PBL driven undergraduate medical program to address medical students' learning and wellbeing.

## Supplementary Information

Supplementary Material 1 (140 KB PDF)
