# Supplementary material for: Mixed method evaluation of a novel seminar format for a PBL-based integrated curriculum
Source: Med Sci Educ. 2026 Apr 9;36(3):1329–40. doi: 10.1007/s40670-026-02708-5 (PMC13356172; doi:10.1007/s40670-026-02708-5)
Supplement: Supplementary file 1 — Supplementary Material 1 (140 KB PDF) [file 40670_2026_2708_MOESM1_ESM.docx]

nCuSP Questionnaire

Project title: Mixed method evaluation of a novel Curtin Seminar Praxis (nCuSP) and usual seminars

This questionnaire has approval from Curtin Human Research Ethics Committee (approval HRE2023-0374)

Your informed consent to participate is required. A Participant Information Statement has been provided in soft copy on Blackboard alongside the seminar resources, and in hard copy at the start of the Seminar. Please alert the tutor if you have not received this Statement. Once you have read it, please confirm whether you consent as follows:
- I have read the Participant Information Statement and I understand its contents
- I believe I understand the purpose, extent and possible risks of my involvement in this research project
- I voluntarily consent to take part in this research project
- I have had an opportunity to ask questions and I am satisfied with the answers I have received
- I understand that this project has been approved by Curtin University Human Research Ethics Committee and will be carried out in line with the National Statement on Ethics Conduct in Human Research (2007)
- I understand that I will keep a copy of the Information Statement

Q1 Please indicate if you will consent:

- Yes. I consent to participate. (1)
- No. I do not consent to participate. (2)

Skip To: End of Survey If Project title: Evaluation of a novel [Anon] Seminar Praxis (nCuSP). .. = No. I do not consent to participate.

| Page Break |  |
| --- | --- |

Q2 Thank you for participating in this research. Please confirm your Year level

- Year 1 (1)
- Year 2 (2)

Display This Question:

If Thank you for participating in this research. Please confirm your Year level... = Year 1

Q3 Which seminar are you providing feedback on?

- Monday 14 August "Intro to Bullying, Harassment and Discrimination" (1)
- Monday 21 August "Pathology of the Brain" (2)
- Monday 2 October "Eating Disorders and Malabsorption" (3)
- Monday 9 October "Fertility / Infertility" (4)

Display This Question:

If Thank you for participating in this research. Please confirm your Year level = Year 2

Q4 Which seminar are you providing feedback on?

- Thursday 7 September "Population Genetics" (1)
- Monday 18 September "Healing and Repair" (2)
- Thursday 21 September "Midwifery, lactation and pregnancy loss" (3)
- Monday 9 October "Bullying, Harassment and Discrimination" (4)

Q5 Please select your response to the following statements

|  | Strongly disagree (1) | Disagree (2) | Neither agree nor disagree (3) | Agree (4) | Strongly agree (5) |
| --- | --- | --- | --- | --- | --- |
| I was confident to engage with the tutor on content (1) |  |  |  |  |  |
| Tutors gave pertinent feedback if I had questions (2) |  |  |  |  |  |
| This seminar developed my understanding of important concepts (3) |  |  |  |  |  |
| The discussion with tutors consolidated my learning (4) |  |  |  |  |  |
| The discussion with tutors helped me to think critically (5) |  |  |  |  |  |
| This seminar helped my studying to be time efficient (6) |  |  |  |  |  |
| This seminar increased my motivation to study (7) |  |  |  |  |  |
| I am satisfied with my learning experience (8) |  |  |  |  |  |

Display This Question:

If Which seminar are you providing feedback on? = Monday 2 October "Eating Disorders and Malabsorption"

Or Which seminar are you providing feedback on? = Monday 9 October "Fertility / Infertility"

Or Which seminar are you providing feedback on? = Monday 18 September "Healing and Repair"

Or Which seminar are you providing feedback on? = Monday 9 October "Bullying, Harassment and Discrimination"

Q6 Please select your response to the following statements

|  | Strongly disagree (1) | Disagree (2) | Neither agree nor disagree (3) | Agree (4) | Strongly agree (5) |
| --- | --- | --- | --- | --- | --- |
| Viewing videos was a better experience than usual seminars (1) |  |  |  |  |  |
| I was aware of content before this seminar began (2) |  |  |  |  |  |
| I had sufficient guidance to complete the clinical vignette (3) |  |  |  |  |  |
| I came prepared with relevant questions (4) |  |  |  |  |  |
| Tutors paid more attention to students in this seminar than in usual seminars (5) |  |  |  |  |  |
| The discussion with fellow students consolidated my learning (6) |  |  |  |  |  |
| The discussion with fellow students promoted my problem-solving abilities (7) |  |  |  |  |  |
| This seminar was easier than usual seminars (8) |  |  |  |  |  |
| I prefer this seminar format to usual seminars for improving knowledge (9) |  |  |  |  |  |

| Page Break |  |
| --- | --- |

Q7 Please tell us about any aspects of your experience of the seminar which were important for your learning

________________________________________________________________

________________________________________________________________

Q8 Please tell us about any aspects of your experience of the seminar which you think should be changed.

________________________________________________________________

________________________________________________________________

Thank you for your response!

________________________________________________________________
